# Supplementary material for: Unlocking global carbon reduction potential by embracing low-carbon lifestyles
Source: Nat Commun. 2025 May 17;16:4599. doi: 10.1038/s41467-025-59269-1 (PMC12085613; doi:10.1038/s41467-025-59269-1)
Supplement: Supplementary file 1 — Supplementary Information [file 41467_2025_59269_MOESM1_ESM.pdf]

Supplementary Information for

Unlocking global carbon reduction potential by embracing low-carbon lifestyles

Yuru Guan [1,2], Yuli Shan [2,3], Ye Hang [2], Qingyun Nie [4], Yu Liu [5,6], Klaus Hubacek [1]**

1. Integrated Research on Energy, Environment and Society (IREES), Energy and Sustainability Research Institute Groningen, University of Groningen, Groningen 9747 AG, the Netherlands
2. School of Geography, Earth and Environmental Sciences, University of Birmingham, Birmingham B15 2TT, UK
3. Birmingham Institute for Sustainability and Climate Action, University of Birmingham, Birmingham B15 2TT, UK
4. School of Management, Nanjing University of Posts and Telecommunications, Nanjing 210003, China
5. College of Urban and Environmental Sciences, Peking University, Beijing 100871, China
6. Institute of Carbon Neutrality, Peking University, Beijing 100871, China

* Corresponding authors: y.shan@bham.ac.uk (Y.S.) and k.hubacek@rug.nl (K.H.)

Contents

1. Supplementary Figures

Supplementary Fig. 1. Global carbon reduction potentials of 21 low-carbon expenditures by four greenhouse gases.

Supplementary Fig. 2. Country-specific GHG savings from expenditure reductions in six categories.

Supplementary Fig. 3. Relative carbon reduction potentials of households from lifestyle changes in 116 countries.

Supplementary Fig. 4. The rebound effects across 21 distinct low-carbon expenditures in regions.

Supplementary Fig. 5. Indirect rebound effect under SC1-SC3 in 116 countries.

Supplementary Fig. 6. Differences in emissions reduction potentials across countries and low-carbon expenditures.

Supplementary Fig. 7. Switzerland food-related carbon footprints and reduction potential from two low-carbon expenditures across five income groups in 2017.

Supplementary Fig. 8. Carbon footprints and reduction potential from low-carbon expenditures across population deciles of Chinese households in 2017.

2. Supplementary Methods: Limitations and Verification of Results

Sensitivity analysis

Cross-verification: physical units vs. monetary units

Switzerland case study

China case study

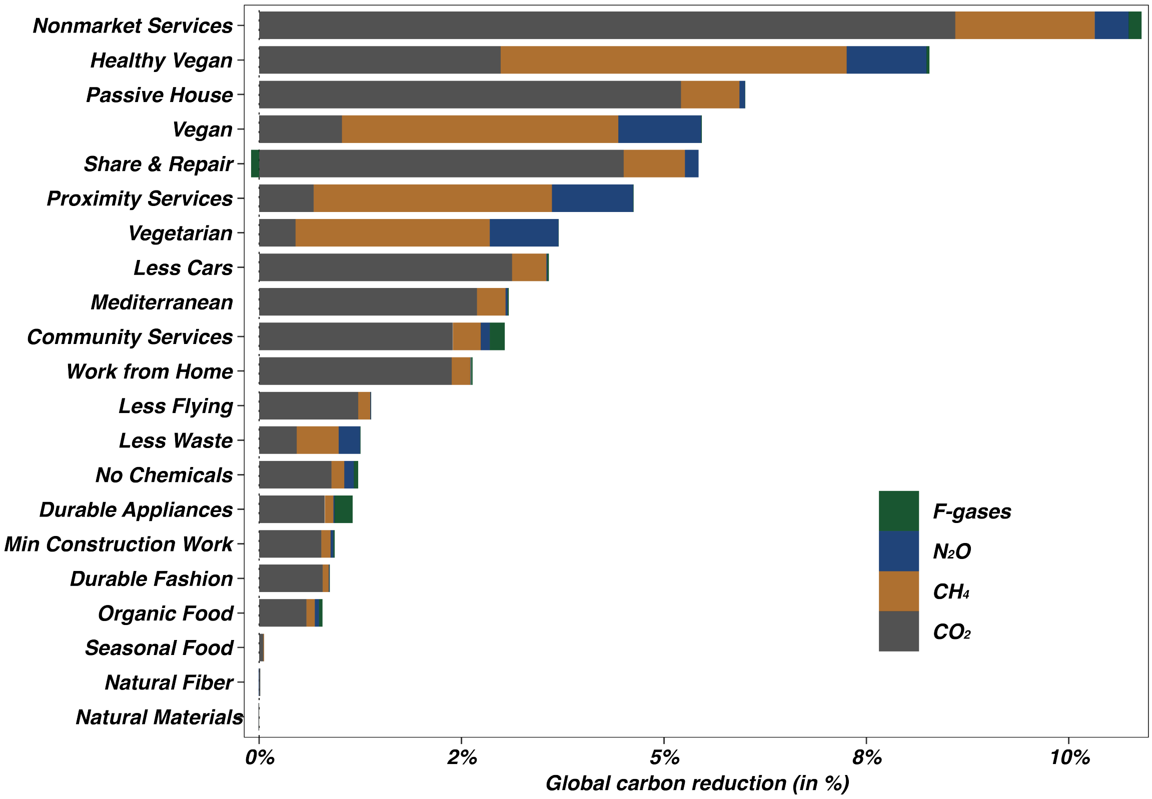

Supplementary Fig. 1. Global carbon reduction potentials of 21 low-carbon expenditures by four greenhouse gases. *Overlaps between different expenditure-led measures and potential rebound effects are not considered in this figure.*

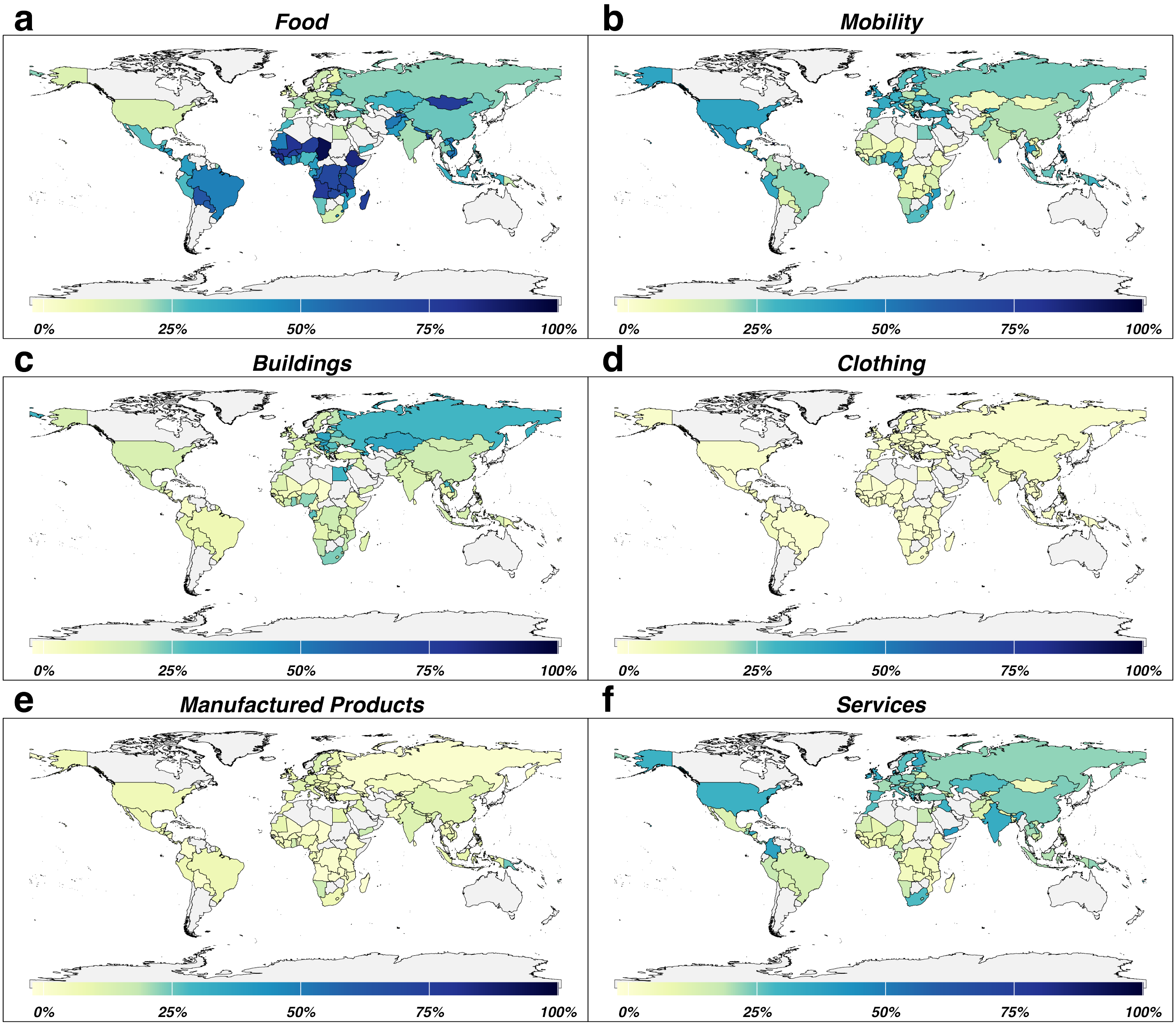

Supplementary Fig. 2. Country-specific GHG savings from expenditure reductions in six categories. *The colours of the map* ***a-f*** *show GHG reduction potentials in six categories from choosing a combination of lifestyle changes across 116 countries. The basemap layer is derived from Runfola, D. et al. geoBoundaries: A global database of political administrative boundaries. PloS one 15, e0231866 (2020), published under the CC BY 4.0 license.*

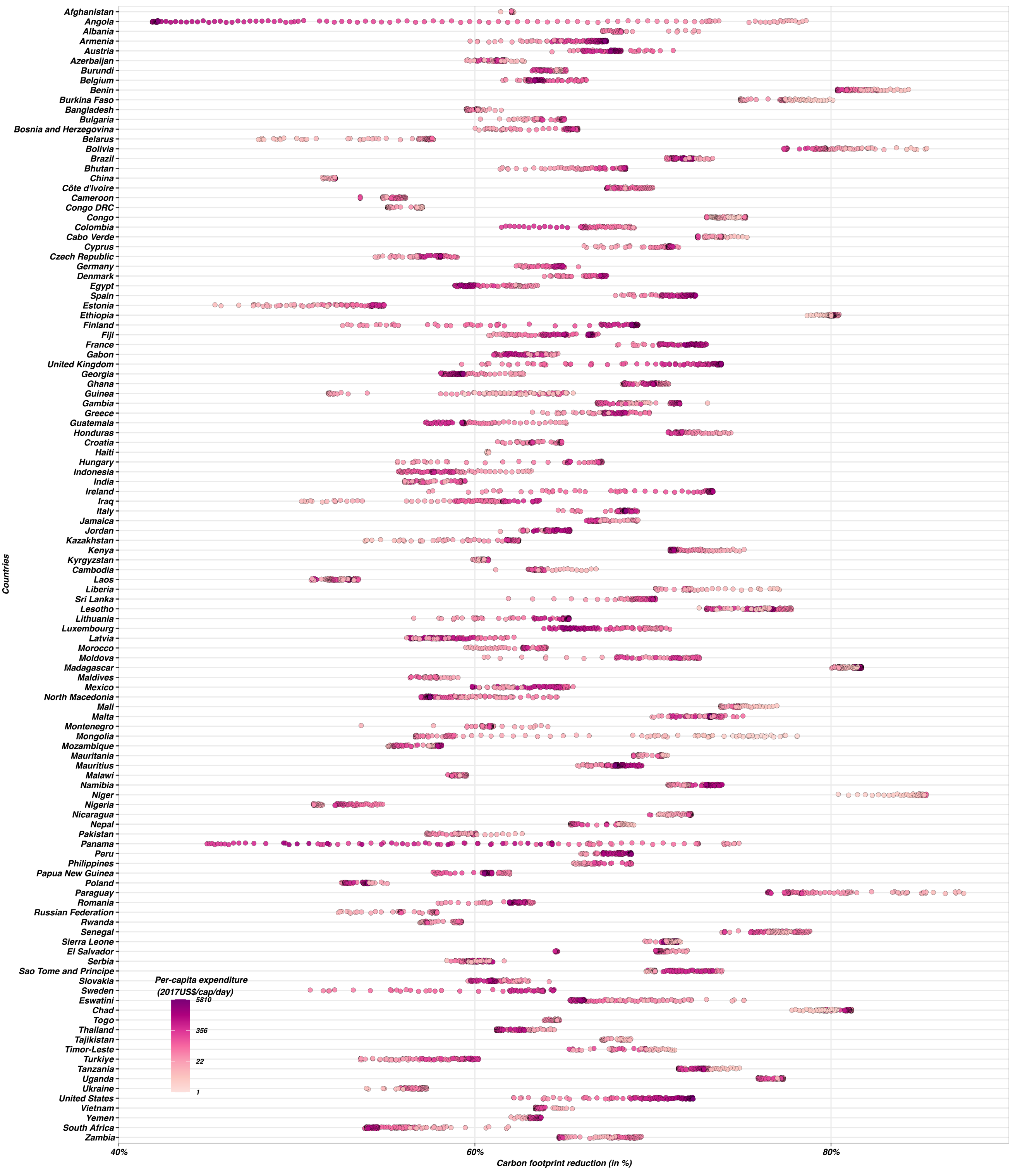

Supplementary Fig. 3. Relative carbon reduction potentials of households from lifestyle changes in 116 countries. *The x-axis displays the relative carbon reduction potentials of household groups, indicating the reduction potential of each household group in relation to its 2017 baseline carbon footprint.*

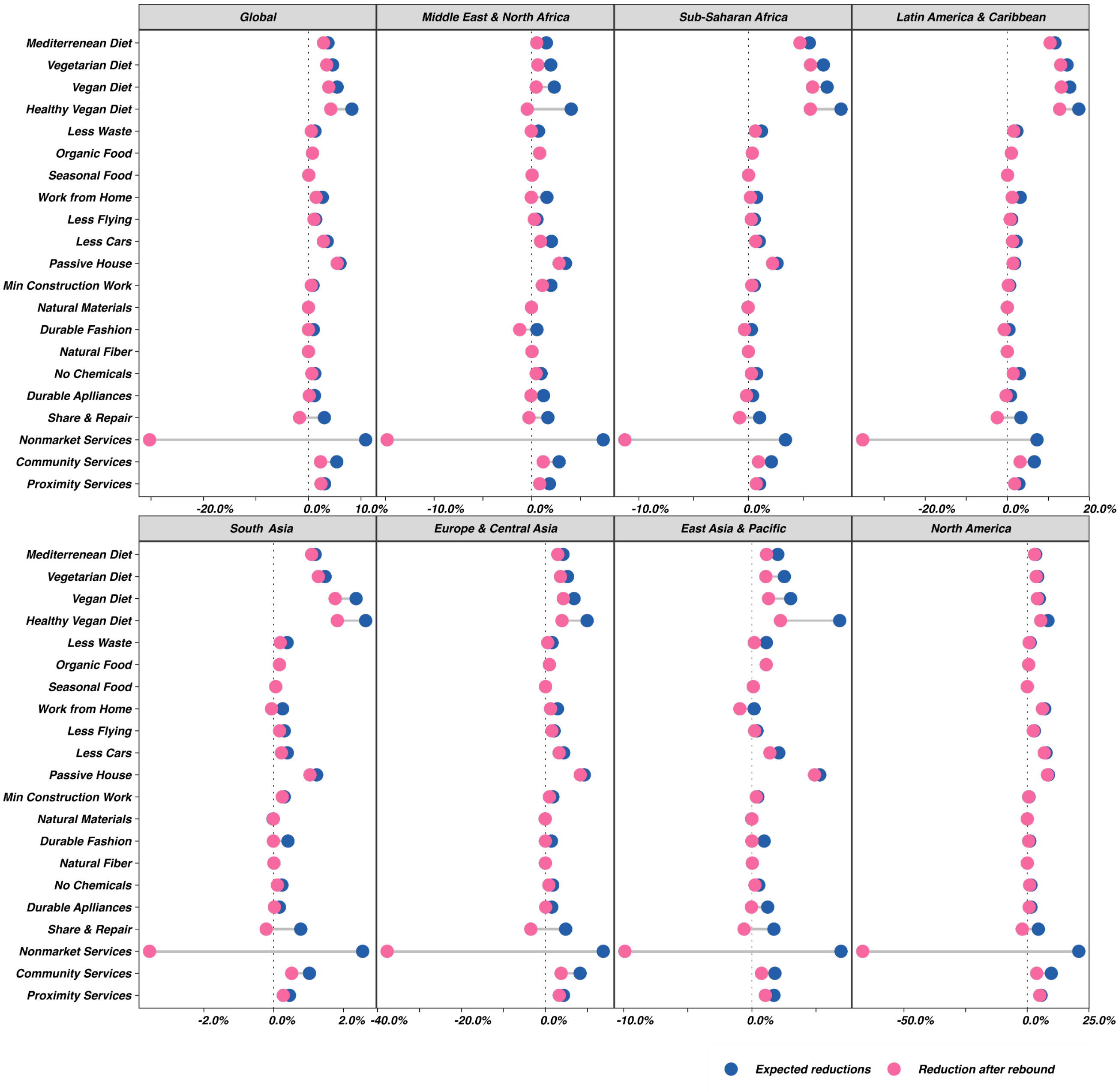

Supplementary Fig. 4. The rebound effects across 21 distinct low-carbon expenditures in regions.

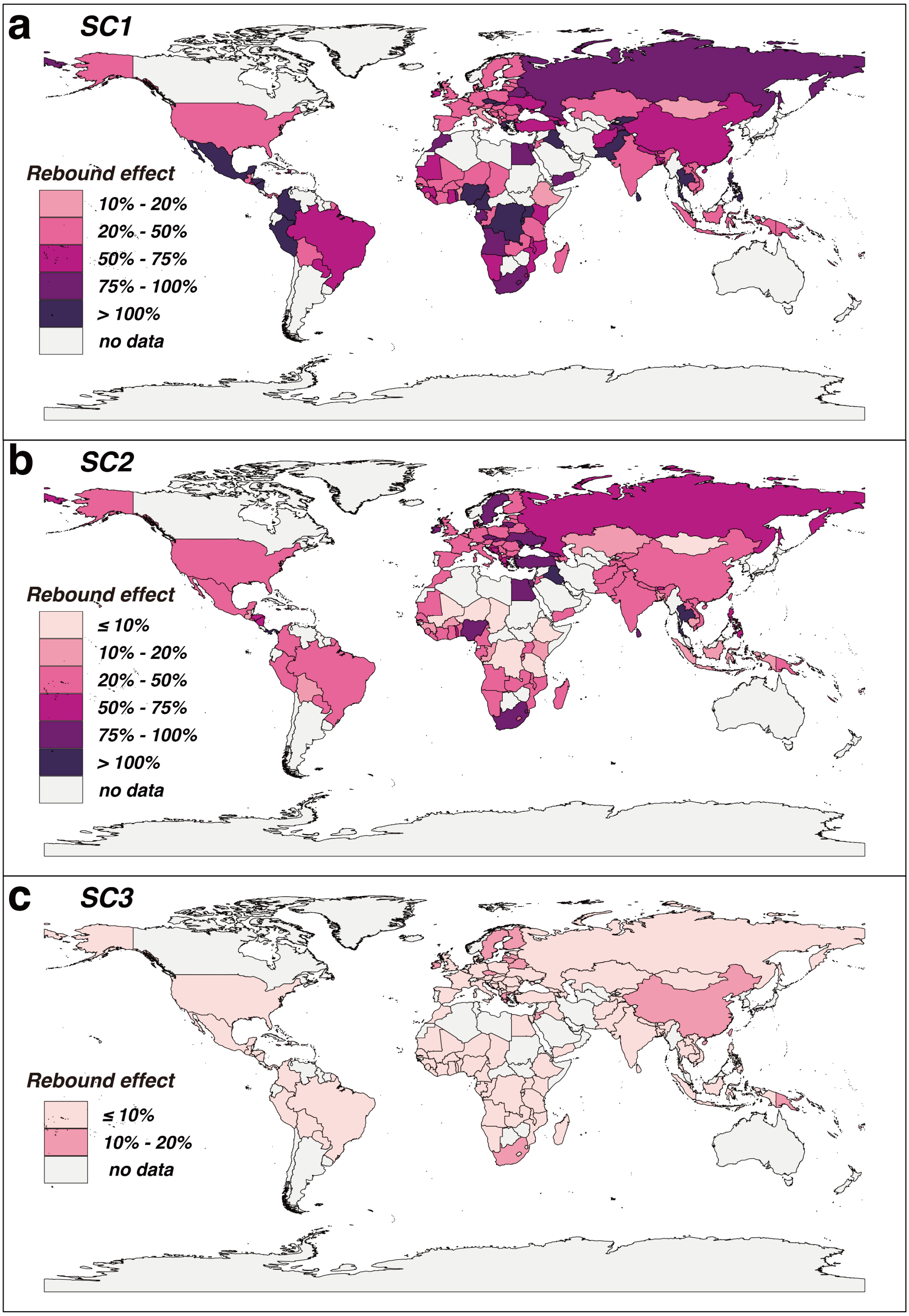

Supplementary Fig. 5. Indirect rebound effect under SC1-SC3 in 116 countries. *In subplot* ***a-c****, the rebound effect is defined as the ratio of offsetting carbon reductions resulting from re-spending saved money to expected reductions under three scenarios. The basemap layer is derived from Runfola, D. et al. geoBoundaries: A global database of political administrative boundaries. PloS one 15, e0231866 (2020), published under the CC BY 4.0 license.*

Supplementary Methods: Limitations and Verification of Results

A key limitation of this study is its reliance on household expenditure surveys combined with Environmentally Extended Multi-Regional Input-Output (EEMRIO) analysis. The EE-IOA framework assumes a linear relationship between spending and carbon footprints, and it cannot capture price and product heterogeneity within national population groups. When applied to sub-groups (derived from household expenditure surveys) that differ significantly from average consumers, it may overestimate the carbon footprint of wealthy individuals due to the assumption that spending directly correlates with emissions. In reality, there is significant variation in the carbon intensity of products and services within each expenditure category. For example, a wealthy individual's purchase of a luxury product may disproportionately increase their carbon footprint compared to a lower-income individual buying a similar, less expensive product. This could lead to an overestimation of the carbon emissions of luxury items, which could inflate the estimated potential for GHG emissions reductions in our analysis.

Sensitivity analysis

We conducted a sensitivity analysis comparing our household-specific EEMRIO database with national-level EEMRIO tables with aggregated final household demand. For the household-specific model, carbon savings are achieved by carbon-exceeding households, and we then aggregated household-level emission reductions to derive the national-level reduction potential. The national-level model assumes a homogeneous population with identical consumption patterns, meaning that all households within a country are treated as “average consumers” with the same carbon footprint. We assumed that if the same changes were made by the corresponding proportion of "average consumers" in each country using national-level MRIO models. Comparing these national-level results allows us to assess the impact of differentiating households based on expenditure levels, as significant deviations for carbon-exceeding households highlight consumption heterogeneity.

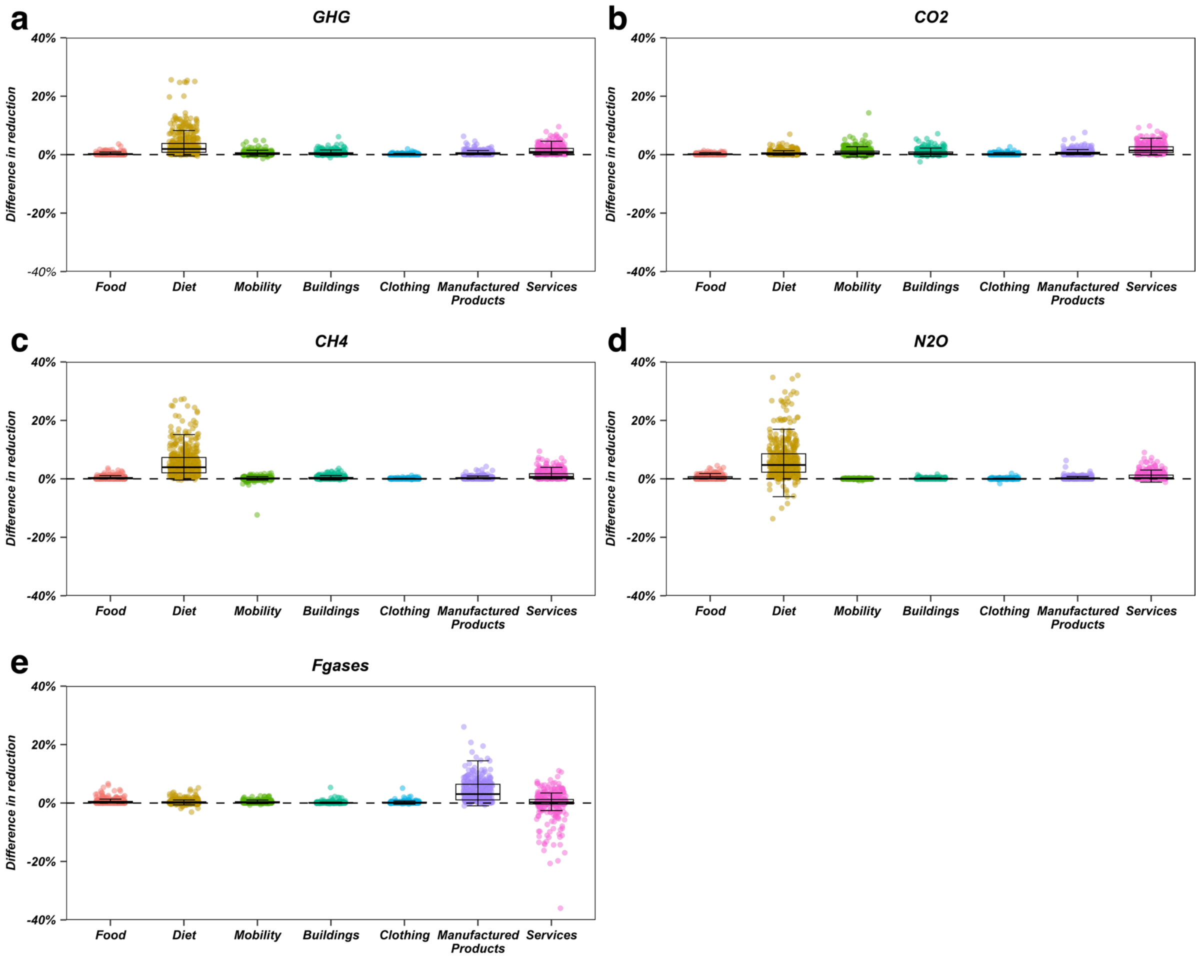

Supplementary Fig. 6. Differences in emissions reduction potentials across countries and low-carbon expenditures. *The Y-axis shows reduction potentials between the household-specific MRIO database with national-level MRIO tables with aggregated final household demand.* ***a-e*** *show reduction difference in GHG, CO_2, CH_4, N_2O, and F-gases.*

As illustrated in Supplementary Fig. 6, the differences in GHG-saving potential between the two models range from -1.4% to 25.6% across various countries and lifestyle measures, with 96% of these differences less than 5% and 99% of them less than 10%. This indicates a relatively high degree of consistency between the models, suggesting that our household-specific model provides a reliable estimate of emission reduction potentials. While the model may slightly overestimate the carbon footprints of high-consumption individuals, particularly in the context of diet-related measures, the overall trends and patterns identified in the study are robust. This overestimation of diet-related measures is likely attributable to the consumption of processed foods, imported products, and luxury food items by wealthier people.

Cross-verification: physical units vs. monetary units

To address uncertainties arising from price heterogeneity within sub-population groups, we cross-validated carbon footprint calculations using physical consumption data (e.g., quantities consumed) [1–3]. This approach was applied to household-level data from Switzerland and China, allowing us to assess carbon footprints and reduction potentials under illustrative low-carbon expenditure scenarios.

Switzerland case study

Switzerland provides highly detailed household expenditure data via its Household Budget Survey (HBS) [3]. The HBS dataset for 2015-2017 includes information on both physical consumption and monetary expenditure data for 105 food products across five income groups (monthly income ranging from 0 to 12,856 Swiss francs)[4].

To calculate food-related carbon footprints for each income group using both physical and monetary units, we linked the HBS data with Switzerland's household final demand data from the GTAP MRIO dataset. The process, using physical consumption data, involved the following steps:

Step1: Generating Consumption Structure Data: we used physical consumption data from the HBS (e.g., rice consumption in kilograms across five income groups), to calculate proportionate shares for each food item across five income groups. This standardization provides a clear picture of how national consumption for each food product is distributed among different income groups.

Step2: Mapping HBS Products to GTAP Sectors: we developed a bridging matrix (Supplementary Table 4) to link these 105 food products in the HBS to the 18 food-related sectors within the GTAP dataset, adhering to the sector definitions from the Swiss Federal Statistical Office and existing studies [3,4]. This allowed us to map the standardized consumption structure data from Step 1 into the 18 GTAP sectors.

Step3: Disaggregating GTAP household Final Demand: Using the mapped consumption structure data generated in Step 2), we disaggregated the GTAP MRIO household final demand for Switzerland across each of the food-related sectors into the five income groups.

Step4: Carbon Footprint Calculation: The disaggregated final demand data were used to calculate the carbon footprints for each income group following the same approach outlined in the main text (Baseline household carbon footprints).

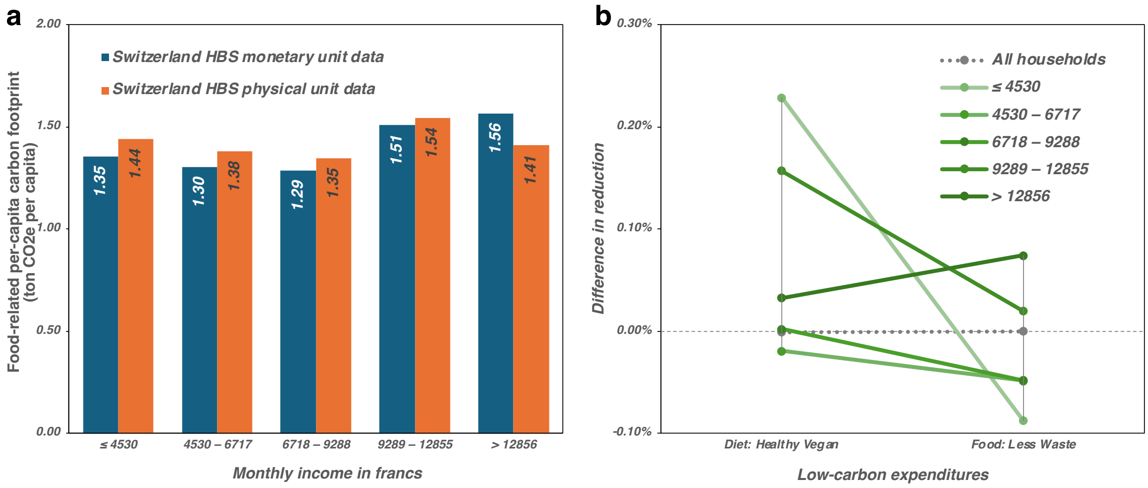

Supplementary Fig. 7. Switzerland food-related carbon footprints and reduction potential from two low-carbon expenditures across five income groups in 2017. *The subplot **a** shows food-related carbon footprints across five income groups in 2017. The subplot **b** compares the reduction potentials derived from monetary-based and physical-based calculations.*

Supplementary Fig. 7 compares the carbon footprint and reduction potential derived from monetary-based and physical-based calculations. Carbon footprints calculated with monetary data ranged from 1.35–1.56 tons CO_2e per capita per year, whereas physical data produced slightly narrower estimates of 1.44–1.54 tons CO_2e per capita per year. Monetary data tend to slightly overestimate footprints for wealthier households and underestimate them for lower-income households compared to physical data. However, the overall differences are small, with percentage deviations (relative to physical data-based results) ranging from -6.0% to 11.0%.

Under two low-carbon food expenditure scenarios—1) adopting a healthy vegan diet and 2) reducing food waste—both calculation methods produced highly consistent carbon savings estimates, reinforcing the robustness of our approach.

China case study

China, a rapidly developing country with considerable variation in population expenditure levels, was selected as a case study. We utilized the 2014 China Household Survey (CHS), which was collected by the National Bureau of Statistics of China using a stratified multistage random sampling approach [5]. Formerly known as China's Urban/Rural Household Income and Expenditure Survey (UHIES/RHIES), it was integrated into a unified urban-rural survey after 2013 [5]. The 2014 CHS dataset used in this study includes over 13,000 households across four provinces (Liaoning, Sichuan, Guangdong, and Shanghai). The dataset provides detailed household-level data on demographics (e.g., size, income, dwelling characteristics) and expenditures for 145 products, 59 of which include physical consumption data (e.g., food, electricity, and private cars). A number of studies have recently used the household consumption survey data from this dataset [6,7].

We grouped China's household survey data into deciles based on their expenditure levels. These subgroup consumption patterns were then bridged with China's household final demand data from the GTAP MRIO data to calculate carbon footprints for each expenditure group in physical and monetary units. The data processing and calculation followed a similar approach as we used for Switzerland. For products lacking physical data, such as school accommodation fees, monetary expenditures were used as proxies for physical consumption, as is commonly used in the literature [3,7]

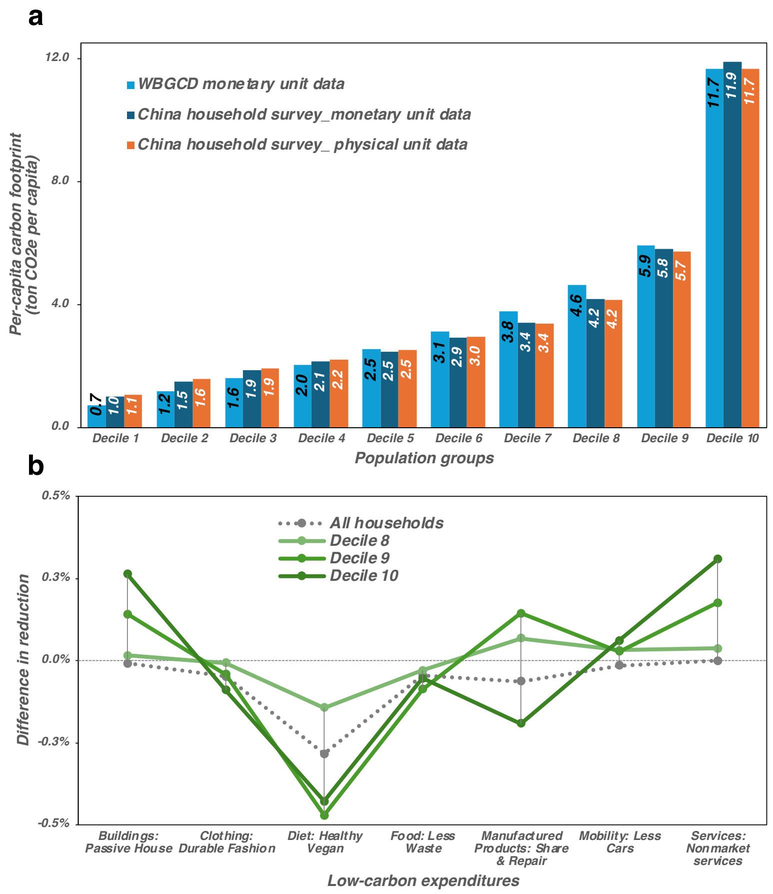

***Supplementary Fig. 8. Carbon footprints and reduction potential from low-carbon expenditures across population deciles of Chinese households in 2017.** The subplot **a** shows household carbon footprints across ten population groups in 2017. The subplot **b** compares the reduction potentials derived from monetary-based and physical-based calculations*

As shown in Supplementary Fig. 8, carbon footprints using monetary data ranged from 1.0 to 11.9 tons CO_2e per capita per year, while physical data produced similar estimates of 1.1 to 11.7 tons CO_2e per capita per year, ranging from -7.6% to 1.9%. As in the Switzerland case study, monetary data slightly overestimates footprints for higher-income households and underestimates them for lower-income households. Despite these small discrepancies the overall trends and patterns remain consistent. By comparing results using monetary data from the modified World Bank Global Consumption Dataset (WBGCD) in the main text (0.7 to 11.7 tons CO_2e per capita per year) with those derived from the China Household Survey data, we conclude the approach used in this study provides robust estimates of carbon footprints.

We analysed seven low-carbon expenditure scenarios for carbon-exceeding households (the same population proportions as in the main text) including areas in Buildings (Passive House), Clothing (Durable Fashion), Diet (Healthy Vegan), Food (Less Waste), Manufactured Products (Share & Repair), Mobility (Fewer Cars), and Services (Nonmarket Services). The results were consistent across both physical and monetary units, demonstrating the robustness of our methodology.

Supplementary References

1. Kilian, L., Owen, A., Newing, A. & Ivanova, D. Microdata selection for estimating household consumption-based emissions. *Economic Systems Research* **35**, 325–353 (2023).

2. André, M., Bourgeois, A., Combet, E., Lequien, M. & Pottier, A. Challenges in measuring the distribution of carbon footprints: The role of product and price heterogeneity. *Ecological Economics* **220**, 108122 (2024).

3. Girod, B. & De Haan, P. More or Better? A Model for Changes in Household Greenhouse Gas Emissions due to Higher Income. *Journal of Industrial Ecology* **14**, 31–49 (2010).

4. Switzerland Federal Statistical Office. Switzerland Household Budget Survey, 2015-2017. https://www.bfs.admin.ch/bfs/en/home/statistiken/wirtschaftliche-soziale-situation-bevoelkerung/einkommen-verbrauch-vermoegen/haushaltsbudget.html (2024).

5. National Bureau of Statistics. Information on China Household Survey. *Household Survey* https://www.stats.gov.cn/hd/cjwtjd/202302/t20230207_1902268.html (2023).

6. Zha, D., Su, X. & Al-Samhi, M. M. M. Will rebound behaviour diminish the decarbonization potential of carbon generalized system of preferences in China? *Sustainable Production and Consumption* **47**, 474–484 (2024).

7. Zhang, Y., Wang, F. & Zhang, B. The impacts of household structure transitions on household carbon emissions in China. *Ecological Economics* **206**, 107734 (2023).
