# Supplementary material for: Unlocking global carbon reduction potential by embracing low-carbon lifestyles
Source: Nat Commun. 2025 May 17;16:4599. doi: 10.1038/s41467-025-59269-1 (PMC12085613; doi:10.1038/s41467-025-59269-1)
Supplement: Supplementary file 2 — Description of Addtional Supplementary Files [file 41467_2025_59269_MOESM2_ESM.pdf]

Description of Additional Supplementary files

Supplementary Data 1. Commodity categories.

Supplementary Data 2. Modelling parameters of 21 low-carbon expenditures.

Supplementary Data 3. List of 116 countries and their mitigation potentials from a combination of low-carbon lifestyles.

Supplementary Data 4. Bridging matrix between food products from the Swiss Household Budget Survey and the 18 food-related sectors in GTAP.
