# Supplementary material for: Unlocking global carbon reduction potential by embracing low-carbon lifestyles
Source: Nat Commun. 2025 May 17;16:4599. doi: 10.1038/s41467-025-59269-1 (PMC12085613; doi:10.1038/s41467-025-59269-1)
Supplement: Supplementary file 4 — Reporting Summary [file 41467_2025_59269_MOESM4_ESM.pdf]

nature portfolio

Corresponding author(s): Klaus Hubacek; Yuli Shan

Last updated by author(s): Mar 11, 2025

Reporting Summary

Nature Portfolio wishes to improve the reproducibility of the work that we publish. This form provides structure for consistency and transparency in reporting. For further information on Nature Portfolio policies, see our Editorial Policies and the Editorial Policy Checklist.

Statistics

For all statistical analyses, confirm that the following items are present in the figure legend, table legend, main text, or Methods section.

n/a	Confirmed	
☐	☒	The exact sample size (n) for each experimental group/condition, given as a discrete number and unit of measurement
☒	☐	A statement on whether measurements were taken from distinct samples or whether the same sample was measured repeatedly
☒	☐	The statistical test(s) used AND whether they are one- or two-sided<br>*Only common tests should be described solely by name; describe more complex techniques in the Methods section.*
☒	☐	A description of all covariates tested
☐	☒	A description of any assumptions or corrections, such as tests of normality and adjustment for multiple comparisons
☐	☒	A full description of the statistical parameters including central tendency (e.g. means) or other basic estimates (e.g. regression coefficient) AND variation (e.g. standard deviation) or associated estimates of uncertainty (e.g. confidence intervals)
☒	☐	For null hypothesis testing, the test statistic (e.g. F, t, r) with confidence intervals, effect sizes, degrees of freedom and P value noted<br>*Give P values as exact values whenever suitable.*
☒	☐	For Bayesian analysis, information on the choice of priors and Markov chain Monte Carlo settings
☒	☐	For hierarchical and complex designs, identification of the appropriate level for tests and full reporting of outcomes
☒	☐	Estimates of effect sizes (e.g. Cohen's d, Pearson's r), indicating how they were calculated

Our web collection on statistics for biologists contains articles on many of the points above.

Software and code

Policy information about availability of computer code

Data collection	No specific software was used for data collection. The data sources were gathered from the web, databases, or directly provided by collaborators. Code for linking lifestyle change scenarios to environmentally extended multi-regional input-output tables was partly retrieved from Wood et al. (2018) and https://figshare.com/s/ab87776ce825f6c53c27. The MATLAB scripts of this work are publicly available on Zenodo (10.5281/zenodo.13618883).
Data analysis	Commercial software, Matlab R2024a (license to the University of Groningen) is used for data processing. Public software R (Version 2024.04.2) are used for result visualization.

For manuscripts utilizing custom algorithms or software that are central to the research but not yet described in published literature, software must be made available to editors and reviewers. We strongly encourage code deposition in a community repository (e.g. GitHub). See the Nature Portfolio guidelines for submitting code & software for further information.

Data

Policy information about availability of data

All manuscripts must include a data availability statement. This statement should provide the following information, where applicable:

- Accession codes, unique identifiers, or web links for publicly available datasets
- A description of any restrictions on data availability
- For clinical datasets or third party data, please ensure that the statement adheres to our policy

Global MRIO table is sourced from the Global Trade Analysis Project (GTAP) 11 Data Bases (pre-release version), provided by co-author Yu Liu. GHG emissions data were obtained from the GTAP 11 Data Bases (official Release). The global expenditure data can be collected from the World Bank. The low-carbon expenditure scenarios dataset originated from Vita et al. (2019). All other socioeconomic data (for example, population and GDP) used in this study were obtained from the World Bank. The main results data generated in this study are provided in the main text and Supplementary Data. More detailed results are available from the corresponding author on request.

Research involving human participants, their data, or biological material

Policy information about studies with human participants or human data. See also policy information about sex, gender (identity/presentation), and sexual orientation and race, ethnicity and racism.

Reporting on sex and gender	n/a
Reporting on race, ethnicity, or other socially relevant groupings	n/a
Population characteristics	n/a
Recruitment	n/a
Ethics oversight	n/a

Note that full information on the approval of the study protocol must also be provided in the manuscript.

Field-specific reporting

Please select the one below that is the best fit for your research. If you are not sure, read the appropriate sections before making your selection.

☐ Life sciences ☐ Behavioural & social sciences ☒ Ecological, evolutionary & environmental sciences

For a reference copy of the document with all sections, see nature.com/documents/nr-reporting-summary-flat.pdf

Ecological, evolutionary & environmental sciences study design

All studies must disclose on these points even when the disclosure is negative.

Study description	In this study, we quantify the greenhouse gas (GHG) emissions reduction potential of 21 low-carbon expenditures, using a global multi-regional input-output model nested with detailed household expenditure data. Targeting households exceeding the global per-capita average required to stay below 2 degrees, our model captures changes in direct energy use, household consumption and upstream intermediate industrial inputs.
Research sample	In this study, we use the World Bank's Global Consumption Database in combination with GTAP (version 11) for the year 2017. Our analysis covers 201 expenditure groups across 116 countries across 65 economic sectors, representing 79.5% of global GDP and 87.3% of the global population.
Sampling strategy	The study includes 116 countries which are represented in the World Bank's Global Consumption Database. Each country is categorized into up to 201 expenditure groups based on per capita annual consumption levels, ranging from $0 to $1 million in 2011 Purchasing Power Parity (PPP) terms. These datasets are the most detailed available to date.
Data collection	The Global MRIO table is sourced from the Global Trade Analysis Project (GTAP) 11 Data Base (pre-release version), provided by Yu Liu. GHG emissions data were obtained from the official release of the GTAP 11 Data Base, collected by Yuru Guan under license from Yuli Shan. The global expenditure data were collected from the World Bank by Yuru Guan. All other socioeconomic data (e.g., population and GDP) used in this study were also obtained from the World Bank by Yuru Guan.
Timing and spatial scale	We analyze the GHG emissions and reduction potential for the year 2017 as the baseline. The data collection and analysis starts on 1/12/2022 and ends on 10/11/2024.
Data exclusions	The World Bank's Global Consumption Database covers only 116 countries.

Reproducibility	We provide all the detailed methods and data sources, programming code and results in both the manuscript and supplementary information files to ensure the reproducibility of this work.
Randomization	n/a
Blinding	n/a

Did the study involve field work? ☐ Yes ☒ No

Reporting for specific materials, systems and methods

We require information from authors about some types of materials, experimental systems and methods used in many studies. Here, indicate whether each material, system or method listed is relevant to your study. If you are not sure if a list item applies to your research, read the appropriate section before selecting a response.

Materials & experimental systems

n/a	Involved in the study	
☒	☐	Antibodies
☒	☐	Eukaryotic cell lines
☒	☐	Palaeontology and archaeology
☒	☐	Animals and other organisms
☒	☐	Clinical data
☒	☐	Dual use research of concern
☒	☐	Plants

Methods

n/a	Involved in the study	
☒	☐	ChIP-seq
☒	☐	Flow cytometry
☒	☐	MRI-based neuroimaging

Plants

Seed stocks	n/a
Novel plant genotypes	n/a
Authentication	n/a
